# Pathological Complete Response to Neoadjuvant Chemoimmunotherapy for Early Triple-Negative Breast Cancer: An Updated Meta-Analysis

**DOI:** 10.3390/cells11121857

**Published:** 2022-06-07

**Authors:** Alessandro Rizzo, Antonio Cusmai, Raffaella Massafra, Samantha Bove, Maria Colomba Comes, Annarita Fanizzi, Lucia Rinaldi, Silvana Acquafredda, Gennaro Gadaleta-Caldarola, Donato Oreste, Alfredo Zito, Francesco Giotta, Vito Lorusso, Gennaro Palmiotti

**Affiliations:** 1 Struttura Semplice Dipartimentale di Oncologia Medica per la Presa in Carico Globale del Paziente Oncologico “Don Tonino Bello”, IRCCS, Istituto Tumori “Giovanni Paolo II”, Viale Orazio Flacco 65, 70124 Bari, Italy; antoniocusmai@hotmail.com (A.C.); l.rinaldi@oncologico.bari.it (L.R.); s.acquafredda@oncologico.bari.it (S.A.); gennaropalmiotti@hotmail.it (G.P.); 2 Struttura Semplice Dipartimentale di Fisica Sanitaria, IRCCS, Istituto Tumori “Giovanni Paolo II”, Viale Orazio Flacco 65, 70124 Bari, Italy; massafraraffaella@gmail.com (R.M.); s.bove9@studenti.uniba.it (S.B.); mariac.comes@libero.it (M.C.C.); annarita.fanizzi.af@gmail.com (A.F.); 3 Medical Oncology Unit, ‘Mons. R. Dimiccoli’ Hospital, Barletta (BT), Azienda Sanitaria Locale Barletta, 76121 Barletta, Italy; gergad@libero.it; 4 Radiology Unit, Istituto di Ricovero e Cura a Carattere Scientifico (IRCCS) Giovanni Paolo II, 70124 Bari, Italy; donatoreste@gmail.com; 5 Unità Operativa Complessa di Anatomia Patologica, IRCCS, Istituto Tumori “Giovanni Paolo II”, Viale Orazio Flacco 65, 70124 Bari, Italy; a.zito@oncologico.bari.it; 6 Unità Operativa Complessa di Oncologia Medica, IRCCS, Istituto Tumori “Giovanni Paolo II”, Viale Orazio Flacco 65, 70124 Bari, Italy; francescogiotta@libero.it (F.G.); vitolorusso@me.com (V.L.)

**Keywords:** breast cancer, pembrolizumab, atezolizumab, durvalumab, immunotherapy, neoadjuvant, immune checkpoint inhibitors

## Abstract

Immune checkpoint inhibitors (ICIs) have made a breakthrough in the systemic treatment for metastatic triple-negative breast cancer (TNBC) patients. However, results of phase II and III clinical trials assessing ICIs plus chemotherapy as neoadjuvant treatment were controversial and conflicting. We performed a meta-analysis aimed at assessing the Odds Ratio (OR) of the pathological complete response (pCR) rate in trials assessing neoadjuvant chemoimmunotherapy in TNBC. According to our results, the use of neoadjuvant chemoimmunotherapy was associated with higher pCR (OR 1.95; 95% Confidence Intervals, 1.27–2.99). In addition, we highlighted that this benefit was observed regardless of PD-L1 status since the analysis reported a statistically significant and clinically meaningful benefit in both PD-L1 positive and PD-L1 negative patients. These findings further support the exploration of the role of ICIs plus chemotherapy in early-stage TNBC, given the potentially meaningful clinical impact of these agents. Further studies aimed at more deeply investigating this emerging topic in breast cancer immunotherapy are warranted.

## 1. Introduction

Triple-negative breast cancer (TNBC) represents a clinically aggressive type of malignancy accounting for approximately 15–20% of all breast tumors [1]. TNBC was suggested to be more frequent in young pre-menopausal, Hispanic, African, and American women [2]; of note, if these malignancies are known to be particularly sensitive to cytotoxic chemotherapy, recurrence rates and mortality are higher compared with other subtypes, especially when a complete response is not achieved following neoadjuvant chemotherapy [3]. As these breast cancer cells lack estrogen receptors, progesterone receptors, and epidermal growth factor receptor 2 (HER2) receptors, chemotherapy is considered the standard treatment for TNBC patients [4]. However, recent years have seen the development and emergence of novel treatment options for this disease, including PARP inhibitors, antibody-drug conjugates (ADCs), and immune checkpoint inhibitors (ICIs) [5,6,7]. In fact, gene expression profiling highlighted that approximately 15–20% of TNBC patients carry BRCA1 or BRCA2 gene mutations and/or deficiencies, which play a crucial role in impairing DNA stability and in promoting carcinogenesis [8]. Thus, PARP inhibitors were reported to be effective in this patient population, as witnessed by the results of practice-changing clinical trials exploring olaparib and talazoparib in the metastatic setting [9,10]. As regards ICIs, if modern immunotherapy has revolutionized the treatment landscape of several solid tumors, including metastatic TNBC, its role as part of neoadjuvant therapy remains to be fully elucidated yet [11,12]. From a biological point of view, TNBC represents an immunogenic breast cancer subtype, since a number of preclinical reports have detected the presence of high levels of immune cell infiltrates and high tumor mutational burden in this disease [13].

Several phase II and III trials have recently investigated the role of chemoimmunotherapy in this setting. Among these, two major studies, the KEYNOTE-522 and the IMpassion031, highlighted that the combination of immunotherapy plus chemotherapy significantly improved the pathological complete response (pCR) compared with chemotherapy alone [14]. However, several questions remain open regarding the role of neoadjuvant ICIs, including the identification of reliable predictors of response to TNBC immunotherapy; unfortunately, a non-negligible proportion of patients do not benefit from these approaches, as witnessed by the results of the GeparNuevo and NeoTRIP trials, which failed to meet their primary endpoints [15,16,17,18,19]. Based on these premises, we performed a comprehensive and updated meta-analysis aiming to evaluate the pCR rate in randomized controlled trials (RCTs) assessing ICIs plus chemotherapy for early TNBC patients.

## 2. Materials and Methods

### 2.1. Search Strategy

All phase II and III clinical trials published from 15 June 2008, to 10 April 2022, evaluating neoadjuvant chemoimmunotherapy for TNBC patients with early-stage disease were retrieved by three different authors. Keywords used for searching on EMBASE, Cochrane Library, and PubMed/Medline were the following: “immunotherapy” OR “nivolumab” OR “ipilimumab” OR “atezolizumab” OR “pembrolizumab” OR “durvalumab” OR “avelumab” OR “immune checkpoint inhibitors” AND “chemotherapy” OR “carboplatin” OR “epirubicin” OR “paclitaxel” OR “nab-paclitaxel” OR “anthracyclines” AND “neoadjuvant therapy” OR “neoadjuvant chemotherapy” OR “preoperative treatment” AND “breast cancer” OR “triple negative breast cancer” OR “early stage breast cancer” OR “TNBC”. Only articles written in English language and published in peer-reviewed journals were included. Proceedings of the main international oncological meetings (such as European Society of Medical Oncology [ESMO], American Society of Clinical Oncology [ASCO], American Association for Cancer Research [AACR], European CanCer Organization [ECCO]) were also searched from 2008 onward for relevant trials and/or abstracts.

### 2.2. Selection Criteria

RCTs retrieved from the first analysis we conducted were restricted to (1) prospective phase II and III RCTs in early TNBC patients; (2) subjects receiving neoadjuvant chemoimmunotherapy; (3) studies with available data in terms of pCR rate in the experimental and the control group, using the definition of ypT0/Tis ypN0 at the time of definitive surgery.

### 2.3. Data Extraction

The following data were extracted for each publication: (1) RCT general information, including first author’s name, year, phase; (2) intervention arms and dosage of drugs; (3) number of TNBC patients with early-stage disease; (4) available outcomes in terms of pCR rate in patients receiving chemoimmunotherapy and chemotherapy alone. Available outcomes were measured as Odds Ratios (ORs) and 95% Confidence Intervals (CIs). Three separate authors conducted the search and identification of RCTs independently. The current meta-analysis was conducted according to Preferred Reporting Items for Systematic Review and Meta-Analyses (PRISMA) guidelines (Supplementary Materials) [20].

### 2.4. Risk of Bias Assessment in Included Studies

**Figure 1:**
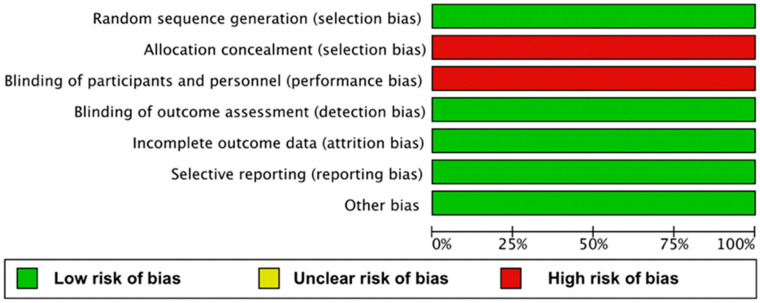
Risk of bias graph; authors’ judgments about each risk of bias item are presented as percentages across all included studies.

The methodological quality of the included studies was assessed by using the Cochrane Collaboration Tool; risk of bias in RCTs was evaluated independently by three separate authors [21]. Studies were examined as having a “low risk”, “high risk”, or “unclear risk” of bias across the specified domains of selection bias, performance bias, attrition bias, and reporting bias. The lists of outcomes reported in the published papers were compared with those from trial registries and study protocols. Any disagreements were resolved by discussion and consensus by three different authors. The results of the assessment were summarized in a risk of bias graph (Figure 1). The current meta-analysis was not registered in PROSPERO.

### 2.5. Statistical Design

All statistical analyses were performed by using the R Studio Software. RRs were used to analyze dichotomous variables, including pCR, in TNBC patients treated with neoadjuvant ICIs plus chemotherapy versus neoadjuvant chemotherapy alone. Forest plots were used to assess ORs. Statistical heterogeneity between the included trials was investigated using the Chi-square test and the I^2^ statistic; substantial heterogeneity was considered to be present when the I^2^ value was greater than 50% or there was a low *p*-value (less than 0.10) in the Chi-square test [22]. When no heterogeneity was found, the fixed effects model was used, while the authors used the random effects model in case of significant heterogeneity.

## 3. Results

### 3.1. Selected Studies

**Figure 2:**
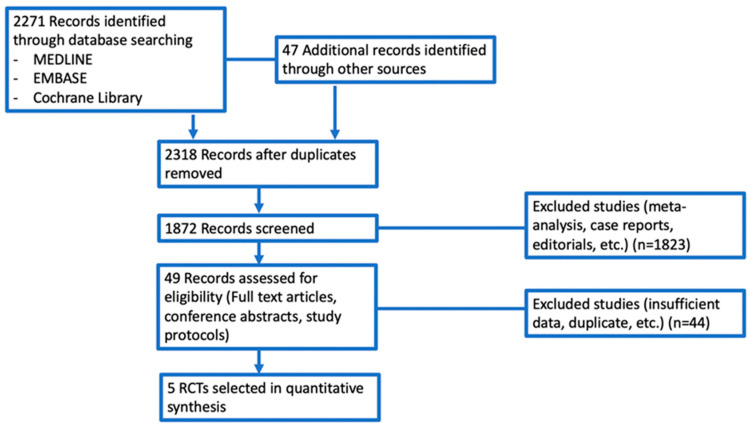
Diagram of all the trials included and excluded in the present meta-analysis. Abbreviations: RCTs—randomized controlled trials.

A total of 2318 potentially relevant reports were identified, which were later restricted to five following an independent evaluation of three authors [15,16,17,18,19]. We excluded 2313 records as non-pertinent reports, including review articles, pre-clinical studies, case reports, editorials, ongoing studies/trials in progress, retrospective studies, systematic review, meta-analyses, single-arm trials, non-randomized trials, etc.). Eligible studies were identified and selected as shown in Figure 2, while a summary of the included RCTs is reported in Table 1. The five studies included in the analysis were RCTs comparing neoadjuvant ICIs plus chemotherapy versus neoadjuvant chemotherapy alone in TNBC patients with early-stage disease [15,16,17,18,19]. A total of 1639 patients (chemoimmunotherapy = 861; chemotherapy alone = 778) were available for the meta-analysis.

### 3.2. Pathological Complete Response Rate

**Figure 3:**
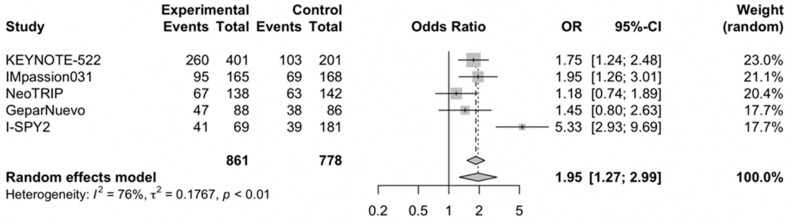
Forest plot of comparison between neoadjuvant chemoimmunotherapy versus chemotherapy alone in early-stage triple-negative breast cancer patients; the outcome of interest was pathological complete response (pCR) rate. Abbreviations: OR: odds ratio.

The pooled OR for the pCR rate in TNBC receiving neoadjuvant chemoimmunotherapy versus chemotherapy alone was 1.95 (95% CI, 1.27–2.99) (Figure 3) [15,16,17,18,19]. The analysis was associated with substantial heterogeneity (I^2^ of 76%), and thus, a random effect model was used.

### 3.3. Pathological Complete Response Rate in PD-L1 Positive and PD-L1 Negative Patients

**Figure 4:**
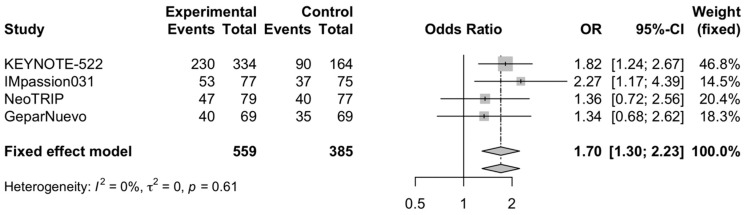
Forest plot of comparison between neoadjuvant chemoimmunotherapy versus chemotherapy alone in early-stage triple-negative breast cancer patients; the outcome of interest was pathological complete response (pCR) rate in PD-L1 positive patients. Abbreviations: OR—odds ratio.

**Figure 5:**
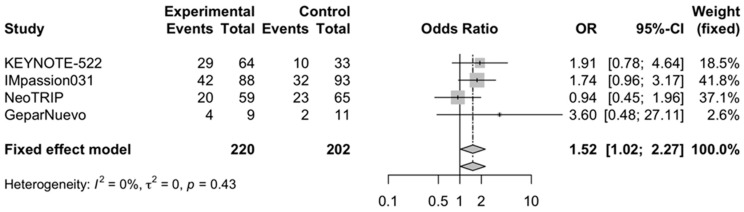
Forest plot of comparison between neoadjuvant chemoimmunotherapy versus chemotherapy alone in early-stage triple-negative breast cancer patients; the outcome of interest was pathological complete response (pCR) rate in PD-L1 negative patients. Abbreviations: OR—odds ratio.

The pooled OR for the pCR rate in PD-L1 positive and PD-L1 negative TNBC receiving neoadjuvant chemoimmunotherapy versus chemotherapy alone was 1.7 (95% CI, 1.3–2.23) (Figure 4) and 1.52 (95% CI, 1.02–2.27) (Figure 5), respectively. Four trials reported specific data regarding the pCR according to PD-L1 status (KEYNOTE-522, IMpassion031, NeoTRIP, and GeparNuevo) [15,16,17,18]. Low heterogeneity was observed, and thus, a fixed-effect model was used for the two analyses.

## 4. Discussion

The last decade has witnessed the emergence of modern immunotherapy, with ICIs, administered as monotherapy or in combination with other anticancer agents such as cytotoxic chemotherapy, targeted drugs, or antiangiogenic agents, making a historical breakthrough in several hematological and solid tumors [23,24,25]. The mechanism of action of anticancer agents acts on different pathways involved in tumor immune escape which is involved in tumor growth. Programmed cell death 1 (PD-1) and cytotoxic T-lymphocyte associated protein 4 (CTLA-4), with their ligands (PD-L1/2 and B7-1/2, respectively), play a pivotal role in this process and represent the main targets of several ICIs [26]. Chemoimmunotherapy has entered everyday clinical practice as a new first-line therapy in metastatic TNBC patients with PD-L1 overexpression or elevated Combined Positive Score (CPS) [27,28]. Based on these premises, ICIs have revolutionized previous TNBC treatment algorithms, prompting researchers and clinicians to consider the expansion of the role of immunotherapy in other settings, including the earlier stage of the disease (e.g., as neoadjuvant and adjuvant therapy). The role of chemoimmunotherapy was assessed in some recently presented and published clinical trials, including the KEYNOTE-522, the IMpassion031, and the GeparNUEVO [29,30]. However, evidence provided by these trials is conflicting, with some positive and negative results reported so far.

Another key point to consider is PD-L1. This biomarker was validated as a predictor of response to chemoimmunotherapy in metastatic BC and has entered everyday clinical practice, following the results of recently published IMpassion130 and KEYNOTE-355 phase III clinical trials. PD-L1 assessment presents some specific methodological issues, including the use of distinct antibodies, scoring systems, and platforms across different studies. The sensitivity and specificity of PD-L1 antibodies are non-superimposable, as also reported in the previously cited randomized controlled trials. Another interesting finding suggesting the non-interchangeable nature of antibodies was highlighted in the KEYNOTE-119 trial. In this study, assessing the PD-1 inhibitor pembrolizumab versus investigator-choice chemotherapy in previously treated metastatic TNBC, one out of ten patients achieving response would have been classified as PD-L1 negative with other scoring systems, such as IC and TPS. In summary, a wide range of challenges and issues are to be considered regarding the assessment of PD-L1 in BC, given the lack of interchangeability between different antibodies, assays, and scoring systems. Moreover, recent studies have also suggested a poor reproducibility among pathologists for IC scoring, and standardization of these methodologies remains a high unmet need in BC immunotherapy.

To the best of the authors’ knowledge, the current meta-analysis represents the most updated and comprehensive study investigating neoadjuvant chemoimmunotherapy in this setting. Our analysis highlighted a higher pCR rate in TNBC patients treated with neoadjuvant chemoimmunotherapy compared with neoadjuvant chemotherapy alone. These findings further support the exploration of the role of ICIs plus chemotherapy in early-stage TNBC, given the potentially meaningful clinical impact of these agents. In addition, we observed that this benefit was reported regardless of PD-L1 status since the analysis suggested a statistically significant and clinically meaningful benefit in both PD-L1 positive and PD-L1 negative patients. However, only a proportion of patients seem to benefit from neoadjuvant chemoimmunotherapy, highlighting the need for a deeper understanding of predictors of response and resistance in this setting.

Some strengths and limitations of our meta-analysis should be highlighted. Among the strengths of this study, our analysis included five phase II and III RCTs by using the most updated data in terms of the pCR rate in the overall population and in the specific subgroups of PD-L1 positive and PD-L1 negative TNBC patients. In addition, we included an overall large number of TNBCs (1639 patients—chemoimmunotherapy = 861, chemotherapy alone = 778). However, some limitations should be underlined. First, the current meta-analysis was based on pooled data, and thus, the presence of single-patient variables was not included. Second, although the random-effects model was performed to reduce heterogeneities across studies, the analysis regarding the pCR in the intention-to-treat population presented substantial heterogeneity; therefore, our findings should be interpreted cautiously. Third, the five trials included in the meta-analysis evaluated widely different combinations. All these agents present different and not superimposable efficacy profiles, and thus, this element could have produced some bias affecting our results. Moreover, it was not possible to simultaneously meta-analyze other potential predictors of response, including novel cytokines, novel surface makers, etc. Lastly, the presence of selection bias cannot be excluded, since all TNBC patients with the early-stage disease were selected subjects enrolled in high-quality trials conducted at academic centers and with good performance status. Despite several limitations that may affect our meta-analysis, we believe these findings could guide in everyday treatment decision-making of TNBC patients receiving neoadjuvant chemoimmunotherapy, also assisting in the design and interpretation of future clinical trials evaluating ICIs in a therapeutic scenario with many unanswered questions.

## 5. Conclusions

**Figure 6:**
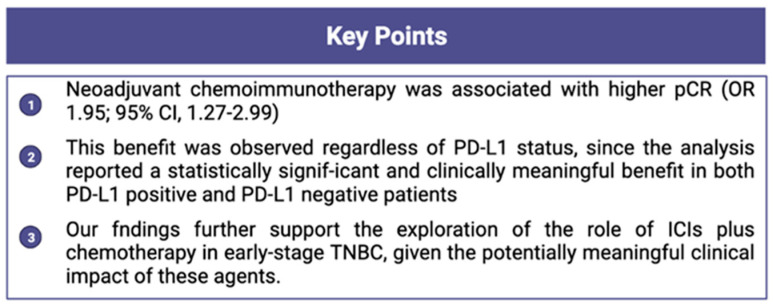
Key points of the current meta-analysis.

The current meta-analysis suggested that the use of neoadjuvant chemoimmunotherapy was associated with a higher pCR rate in TNBC patients compared with neoadjuvant chemotherapy alone, corroborating the results of two large phase III trials (KEYNOTE-522 and IMpassion031) (Figure 6). Further studies aimed at more deeply investigating this emerging topic in breast cancer immunotherapy are awaited. In the current era of medical oncology, progress in this setting will require the collaboration of basic science, novel methodological approaches—such as machine learning and artificial intelligence—and clinical research to optimize systemic treatment and improve the outcomes of TNBC patients treated with neoadjuvant ICIs plus chemotherapy.

## Figures and Tables

**Table 1:** Summary of the included studies. Abbreviations: ICI—immune checkpoint inhibitor.

Trial	Phase	ICI Included	Type of Taxane	Treatment Details
KEYNOTE-522	III	Pembrolizumab	Paclitaxel	- Pembrolizumab plus paclitaxel and carboplatin or placebo plus paclitaxel and carboplatin. - Additional four cycles of pembrolizumab or placebo, and both groups received doxorubicin–cyclophosphamide or epirubicin–cyclophosphamide. - Following definitive surgery, adjuvant pembrolizumab or placebo for up to nine cycles.
IMpassion031	III	Atezolizumab	Nab-paclitaxel	- Nab-paclitaxel for 12 weeks followed by doxorubicin and cyclophosphamide every 2 weeks for 8 weeks plus intravenous atezolizumab or placebo.
NeoTRIP	III	Atezolizumab	Nab-paclitaxel	- Carboplatin and nab-paclitaxel on days 1 and 8, without or with atezolizumab on day 1. - Both regimens were given for eight cycles before surgery followed by four cycles of an adjuvant anthracycline regimen.
GeparNuevo	II	Durvalumab	Nab-paclitaxel	- Initial 2-week window phase in which individuals received intravenous durvalumab 0.75 mg or placebo. - After this, both groups received additional nab-paclitaxel weekly for 12 weeks then epirubicin and cyclophosphamide for 4 cycles.
I-SPY2	II	Pembrolizumab	Paclitaxel	- Neoadjuvant chemotherapy plus pembrolizumab for 4 cycles concurrently with paclitaxel versus intravenous paclitaxel, followed by 4 cycles of doxorubicin plus cyclophosphamide.

## Supplementary Materials

The following supporting information can be downloaded at: https://www.mdpi.com/article/10.3390/cells11121857/s1, Table S1: The PRISMA 2020 statement: an updated guideline for reporting systematic reviews.
